# Modulation of Gut Microbiota Combined with Upregulation of Intestinal Tight Junction Explains Anti-Inflammatory Effect of Corylin on Colitis-Associated Cancer in Mice

**DOI:** 10.3390/ijms23052667

**Published:** 2022-02-28

**Authors:** Zi-Yu Chang, Hsuan-Miao Liu, Yann-Lii Leu, Chung-Hua Hsu, Tzung-Yan Lee

**Affiliations:** 1 Institute of Traditional Medicine, School of Medicine, National Yang Ming Chiao Tung University, Taipei 112304, Taiwan; changzhi887@gmail.com; 2 Graduate Institute of Traditional Chinese Medicine, School of Chinese Medicine, College of Medicine, Chang Gung University, Taoyuan 33302, Taiwan; miaowhale@gmail.com; 3 Graduate Institute of Nature Products, College of Medicine, Chang Gung University, Taoyuan 33305, Taiwan; ylleu@mail.cgu.edu.tw; 4 Tissue Bank, Chang Gung Memorial Hospital, Taoyuan 33305, Taiwan; 5 Department of Traditional Chinese Medicine, Chang Gung Memorial Hospital, Keelung 20401, Taiwan

**Keywords:** inflammatory bowel disease (IBD), corylin, gut microbiota, intestinal tight junction, colorectal cancer

## Abstract

Inflammatory bowel disease (IBD) involves chronic inflammation, loss of epithelial integrity, and gastrointestinal microbiota dysbiosis, resulting in the development of a colon cancer known as colitis-associated colorectal cancer (CAC). In this study, we evaluated the effects of corylin in a mouse model of dextran sodium sulfate (DSS)-induced colitis. The results showed corylin could improved the survival rate and colon length, maintained body weight, and ameliorated the inflammatory response in the colon. Then, we further identified the possible antitumor effects after 30-day treatment of corylin on an azoxymethane (AOM)/DSS-induced CAC mouse model. Biomarkers associated with inflammation, the colon tissue barrier, macrophage polarization (CD11c, CCR7, CD163, and CD206), and microbiota dysbiosis were monitored in the AOM/DSS group versus corylin groups. Corylin downregulated pro-inflammatory cytokines (TNF-α, IFN-γ, IL-1β, and IL-6) mRNA expression and inflammatory signaling-associated markers (TLR4, MyD88, AP-1, CD11b, and F4/80). In addition, a colon barrier experiment revealed that epithelial cell proliferation of the mucus layer (Lgr5, Cyclin D1, and Olfm4) was downregulated and tight junction proteins (claudin-1 and ZO-1) were upregulated. Furthermore, the *Firmicutes*/*Bacteroidetes* ratio changed with corylin intervention, and the microbial diversity and community richness of the AOM/DSS mice were improved by corylin. The comparative analysis of gut microbiota revealed that *Bacteroidetes*, *Patescibacteria*, *Candidatus Saccharimonas*, *Erysipelatoclostridium*, and *Enterorhabdus* were significantly increased but *Firmicutes*, *Turicibacter*, *Romboutsia*, and *Blautia* decreased after corylin treatment. Altogether, corylin administration showed cancer-ameliorating effects by reducing the risk of colitis-associated colon cancer via regulation of inflammation, carcinogenesis, and compositional change of gut microbiota. Therefore, corylin could be a novel, potential health-protective, natural agent against CAC.

## 1. Introduction

Colorectal cancer (CRC) is the most commonly diagnosed malignancy and the leading cause of cancer mortality worldwide [1]. Chronic inflammation has been suggested to be one of the hallmarks of IBD and cancer development. Indeed, a causal relationship between chronic inflammation and CRC carcinogenesis has already been well established [2]. Epidemiological studies have reported that IBD patients exhibit a two- to eight-fold higher CRC risk, and the incidence of CRC can be effectively ameliorated by anti-inflammatory medications [3,4]. Colitis-associated cancer (CAC) represents a relatively ideal model in tumor biology to unravel how inflammatory responses activate tumorigenesis in the normal colonic mucosa [5]. The demand for more effective and safer natural agents to prevent colon cancer has, therefore, increased. Chronic inflammation is thought to contribute to cancer initiation by the persistent release of reactive oxygen and nitrogen species, thus leading to genome damage and carcinogenesis. However, accumulating evidence indicates that inflammatory cells and their products may also play a role in the promotion and progression steps, thus helping cancer cells to grow and acquire new capabilities, such as infiltration and metastasizing. Therefore, there is an urgent need to characterize the possible mechanisms involved in IBD and inflammation-driven colon tumorigenesis. Consequently, it is essential to explore alternative approaches to managing IBD and help prevent CAC.

Gut permeability plays a pivotal role in the maintenance of intestinal homeostasis, metabolism, and immune tolerance. A single epithelial layer of the intestinal barrier consists of multiple components: a robust junctional complex consisting of tight junctions (TJs), which are comprised of multiple strands of adhesive transmembrane molecules (e.g., claudins and occludin), connected via intracellular adaptor proteins (e.g., ZO-1, ZO-2, and ZO-3, cingulin) to the actin cytoskeleton [6,7] to form a physical barrier; goblet cells also secrete mucus that separates the epithelial layer from the luminal microbiota [8]. Disruption of the gut barrier integrity generates a “leaky gut”, allowing an aberrant interaction of the luminal contents with the intestinal mucosal immune system. This process leads to local dysregulation of immune responses, which culminates in chronic inflammation, leading to diseases, such as inflammatory bowel disease (IBD) [9].

Disruption of the gut microbiota or dysbiosis can increase lipopolysaccharide (LPS) production by Gram-negative bacteria, which leads to impaired gut barrier function and causes endotoxemia [10,11,12]. LPS can enter the mesenteric vein, travel into the circulation system, and act on target organs and tissues to cause inflammation and disease [10,13]. On the other hand, one study has shown that gut microbiota are an essential factor in driving inflammation in the colon, and this inflammatory environment is related to CRC development [14]. A recent report suggested that proper modulation of gut microbiota by ingesting specific probiotics may help prevent tumor formation [15]. Furthermore, ingesting prebiotics, which are known to be beneficial to the host by stimulating probiotic bacterial growth, may change the human gut microbiota composition to help specific probiotics for CRC prevention [16]. The relationship between intestinal barrier dysregulation and CAC is confirmed; therefore, finding how to reverse gut microbiota and gut barrier function is a noteworthy strategy in CAC.

Corylin is a compound isolated from the fruit and seed of *Psoralea corylifolia* L. [17] and possesses pharmacological effects due to its antioxidant properties [18] and osteoblastic proliferation-stimulating activity [19]. In addition, corylin is a natural polyphenol compound that has been shown to ameliorate insulin resistance (IR) and liver steatosis [20]. Furthermore, the beneficial effects of corylin in individuals consuming an HFD, as proposed by Chen et al. [20], are that it may reduce the amount of LPS generated by Gram-negative bacteria in the intestine and prevent the deleterious effects of the diet on intestinal epithelial permeability. At present, no study has reported the molecular mechanisms underlying the effects of corylin against colitis-associated cancer, and the possible mechanisms are still largely unknown. The mechanisms involved in leaky gut remain unclear in terms of how corylin reaches the colon and, therefore, interacts with the intestinal microbiome. Furthermore, it has not been determined whether corylin affects the composition of the intestinal flora and ameliorates CAC in AOM/DSS mice.

## 2. Results

### 2.1. Corylin Ameliorated DSS-Induced Colitis and Intestinal Barrier Damage

**Figure 1:**
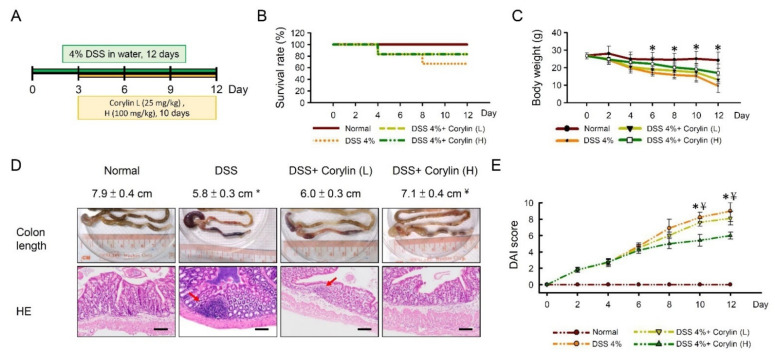

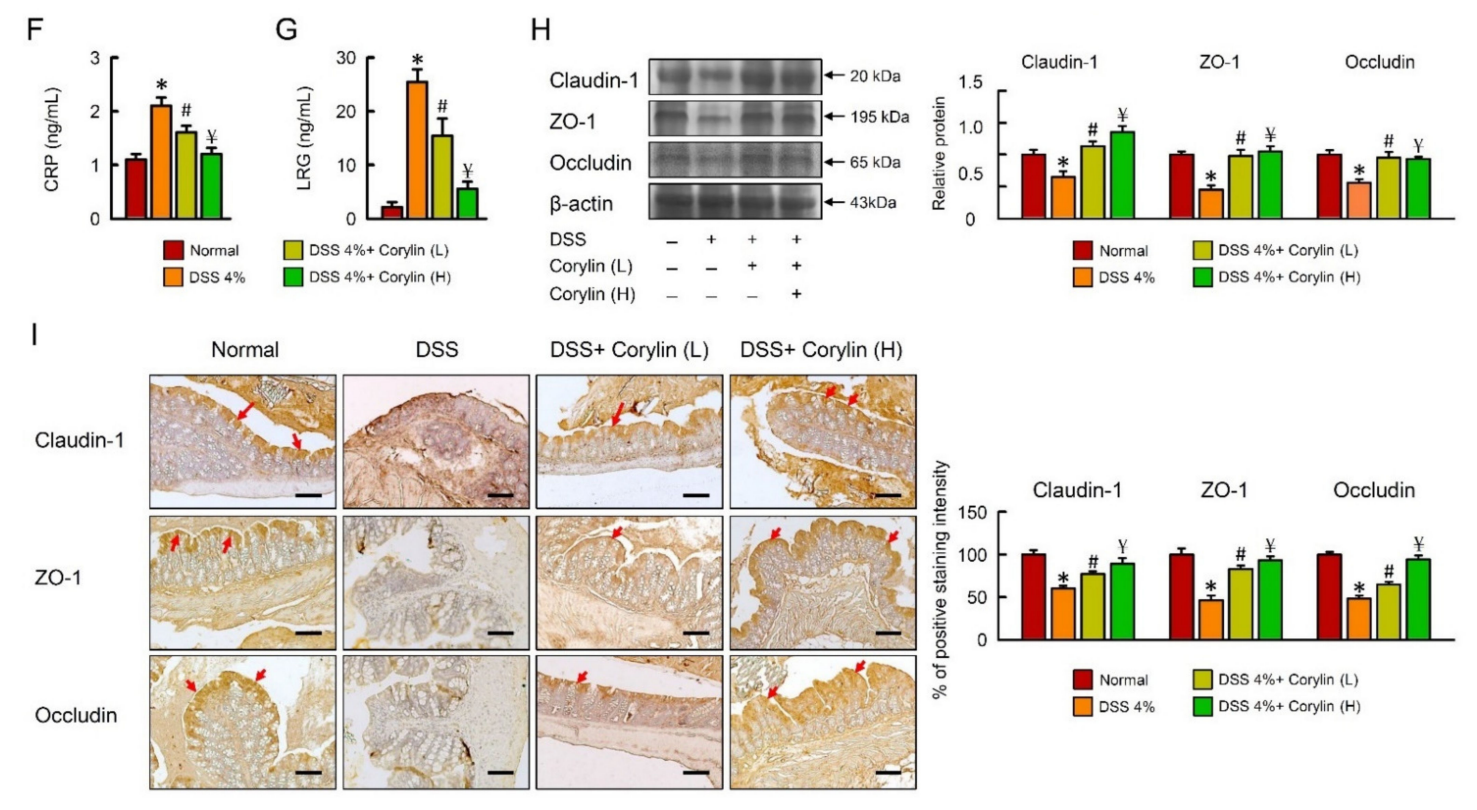
Corylin ameliorates DSS-induced colitis in mice. (**A**) Schematic of the recurring model of DSS-induced ulcerative colitis. (**B**) Survival rate in experimental mice. (**C**) Body weight curves of WT mice treated with 4% DSS and corylin. (**D**) Colon length and HE stain from experimental mice. (**E**) Disease activity index (DAI) score in experimental mice. (**F**) CRP and (**G**) LRG level in experimental mice. (**H**) Western blot analysis of claudin-1, ZO-1, and β-actin (loading control) in colon homogenates. Right graph indicates quantification relative to β-actin. (**I**) Expression analysis by immunohistochemical staining of claudin-1, ZO-1, and occludin in mice after the indicated treatment. Red arrow highlights the positive staining. Scale bar: 100 µm. Tissue staining, as quantified by either stain intensity, is represented on the left-hand vertical axis in each graph. Results represent mean ± SEM. * *p* < 0.05, Normal compared with DSS; ^#^ *p* < 0.05, DSS compared with DSS + Corylin (L); ^¥^ *p* < 0.05, DSS compared with DSS + Corylin (H). DSS, dextran sodium sulfate; ZO-1, zonula occludens-1. HE: hematoxylin and eosin; CRP, C-reactive protein; LRG, leucine-rich alpha 2 glycoprotein.

Upon DSS challenge, mice exhibited a lower survival rate (Figure 1B), increased weight loss (Figure 1C), a shorter colon length (Figure 1D), and a higher disease activity index (DAI) score (Figure 1E) compared with normal mice. When evaluating hematoxylin–eosin-stained slides, these marked histopathological changes in the colon tissues of DSS-treated mice were observed (Figure 1D). Additional, serum C-reactive protein (CRP) and leucine-rich alpha 2 glycoprotein (LRG) were significantly increased in DSS-induced colitis mice (Figure 1F,G). The colons showed more severe inflammation with more cryptitis/crypt abscess formation, erosion, or ulceration in DSS-induced colitis mice compared with normal mice (Figure 1D–G). Compared with the DSS-alone treatment group, the survival rate, body weight, and colon length were significantly increased in the high dose 100 mg/kg (H) corylin treatment groups. In addition, corylin, at a dose of 100 mg/kg (H), significantly reduced DAI, CRP, and LRG (Figure 1B–E).

Furthermore, we confirmed that DSS-induced colitis mice colons showed reduced barrier function. The expression levels of claudin-1, ZO-1, and occludin were significantly decreased in the DSS-alone treatment group. Compared with the DSS-alone treatment group, the expression levels of claudin-1, occludin, and ZO-1 were significantly increased in the corylin treatment groups. Furthermore, corylin, at a dose of 100 mg/kg (H), significantly increased the levels of claudin-1 and occludin, which were even higher than for the effects produced by corylin at a dose of 25 mg/kg (L) compared with colitis mice. We propose that the effects of corylin on DSS-induced colitis mice are related to the regulation of the colonic mucosal barrier function, where the expression levels of claudin-1, ZO-1, and occludin play important roles in maintaining the intestinal mucosal barrier function (Figure 1H,I).

### 2.2. Corylin Attenuated TLR4/p38/AP-1 Signal Pathway and Inflammation in DSS-Induced Colitis Mice

**Figure 2:**
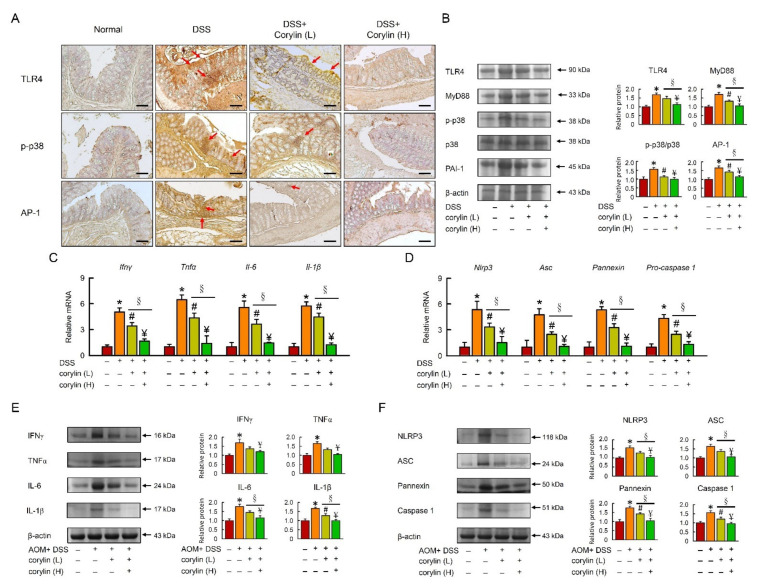
Corylin improves the TLR4 signal pathway in DSS-induced colitis mice. (**A**) Expression analysis by immunohistochemical staining of TLR4, p-p38, and AP-1 in mice after the indicated treatment. Scale bar: 100 μm. Red arrows highlight positive staining. (**B**) Western blot analysis of TLR4, MyD88, p-p38, AP-1, and β-actin (loading control) in colon homogenates. Right graph indicates quantification relative to β-actin. (**C**,**D**) mRNA expression of *Ifnγ*, *Tnf-α*, *Il-6*, *Il-1β*, *Nlrp3*, *Asc*, *Pannexin*, and *Pro-caspase 1* determined by qRT-PCR. (**E**,**F**) Western blot analysis of IFNγ, TNF-α, IL-6, IL-1β, NLRP3, ASC, Pannexin, and Caspase 1, and β-actin (loading control) in colon homogenates. Results represent mean ± SEM. * *p* < 0.05, Normal compared with DSS; ^#^ *p* < 0.05, DSS compared with DSS + Corylin (L); ^¥^ *p* < 0.05, DSS compared with DSS + Corylin (H); ^§^ *p* < 0.05, DSS + Corylin (L) compared with DSS + Corylin (H). DSS, dextran sodium sulfate; TLR4, Toll-like receptor 4; MyD88, myeloid differentiation primary response 88; AP-1, activator protein 1; *Ifnγ*, interferon gamma; *Tnf-α*, tumor necrosis factor-α; *Il-6*, interleukin-6; *Nlrp3*, NACHT, LRR, and PYD domains-containing protein 3.

Growing evidence indicates that the TLR4/NF-κB signaling pathway plays an important role in the pathogenesis of IBD. Thus, targeted suppression of the TLR4 signaling pathway has become a therapeutic strategy for IBD in recent years. To determine the effect of corylin on TLR4 expression, an immunohistochemical analysis and a Western blot analysis were carried out. The immunohistochemical assay showed that DSS markedly increased the overexpression of TLR4, MyD88, p-p38, and AP-1 compared with the normal group. However, the treatment with corylin could decrease signaling on the TLR4 signal pathway (Figure 2A,B).

To investigate the effect of corylin on DSS-induced inflammation and the NLRP3 inflammasome, we further assessed the NLRP3 inflammasome signal. Corylin significantly reduced *Ifnγ*, *Tnf-α*, *Il-6*, *Il-1β*, *Nlrp3*, *Asc*, *Pannexin*, and *Pro-caspase 1* mRNA expression and protein levels in DSS-induced colitis mice. Administration of 100 mg/kg (high dose) corylin significantly reduced the inflammatory response and had a more beneficial effect than 25 mg/kg corylin in DSS-induced colitis mice (Figure 2C–F).

### 2.3. Effects of Corylin on Macrophage Polarization

**Figure 3:**
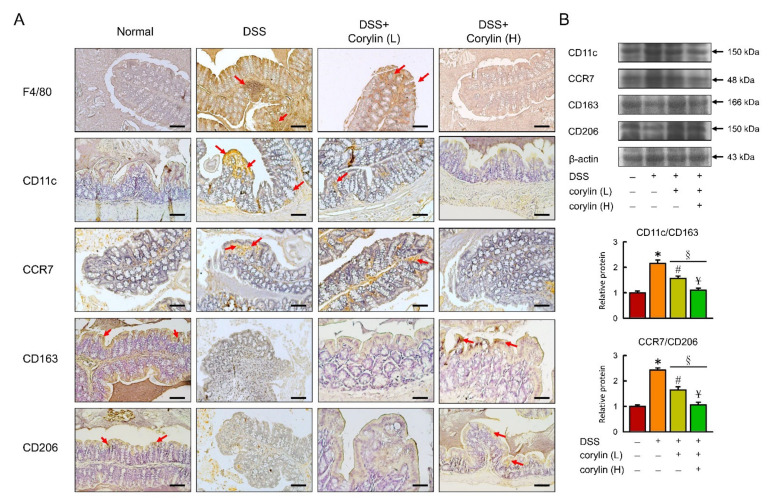
Corylin alters macrophage polarization in DSS-induced colitis mice. (**A**) Representative F4/80, CD11c, CCR7, CD163, and CD206 staining of colon. Red arrows highlight positive staining. Scale bar: 100 μm. (**B**) Above: Western blot analysis of CD11c, CCR7, CD163, CD206, and β-actin (loading control) in colon. Below: Quantification of CD11c, CCR7, CD163, and CD206 protein levels by Western blot in graphs indicating quantification ratio of CD11c and CD163 as well as ratio of CCR7 and CD206 levels. Results represent mean ± SEM. * *p* < 0.05, Normal compared with DSS; ^#^ *p* < 0.05, DSS compared with DSS + Corylin (L); ^¥^ *p* < 0.05, DSS compared with DSS + Corylin (H); ^§^ *p* < 0.05, DSS + Corylin (L) compared with DSS + Corylin (H). DSS, dextran sodium sulfate.

As previous studies have established that M1 macrophages can contribute to the pathogenesis of colitis [21], the possibility that the protective activity of corylin was mediated via an increase in M2 macrophage polarization was examined. Colon tissues were collected from animals treated with DSS for 12 days. M1 macrophages were identified by their expression of Cd11c and CCR7 surface markers, while M2 macrophages were identified by the expression of CD163 and the CD206 mannose receptors, the latter having been established as a reliable marker of M2 macrophages [22]. Corylin caused a significant reduction in M1 macrophage markers and increased M2 macrophage markers (Figure 3A,B). Consistent with previous findings, the inflammation induced by DSS caused the frequency of inflammatory M1 macrophages to increase, and corylin reversed that effect, as shown by changes in the M1/M2 macrophage ratio (Figure 3B).

### 2.4. Effects of Corylin on Stem-like Cell and Intestinal Epithelial Cell Proliferation

**Figure 4:**
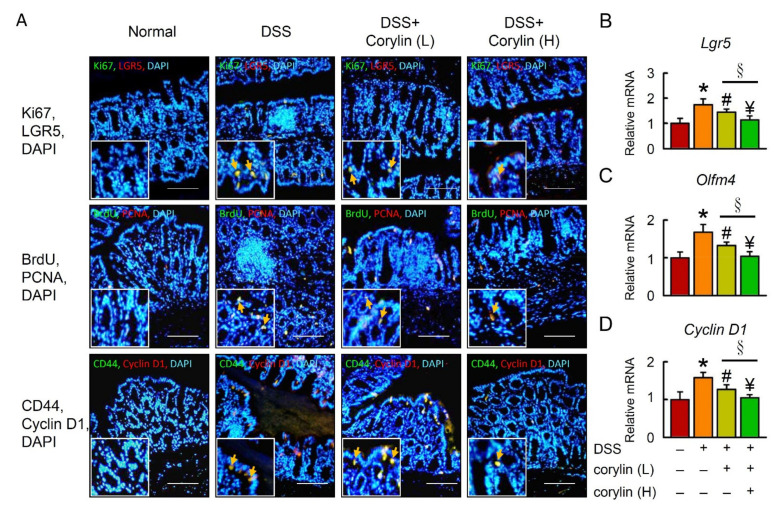
Corylin alters DSS-induced regeneration in mice. (**A**) Top line: Double immunofluorescence staining of Ki67 (green), LGR5 (red), and DAPI (blue) in colon. The staining for Ki67/LGR5 from colon expressing both markers is identified. Middle line: Double immunofluorescence staining of BrdU (green), PCNA (red), and DAPI (blue) in colon. The staining for BrdU/PCNA from colon expressing both markers is identified. Bottom line: Double immunofluorescence staining of endogenous CD44 (green), cyclin D1 (red), and DAPI (blue) in colon. The staining for CD44/cyclin D1 from colon expressing both markers is identified. DAPI (blue). Yellow arrows highlight the positive staining. Scale bar: 100 μm. (**B**–**D**) mRNA expression of *Lgr5*, *Olfm4*, and *Cyclin D1* as determined by qRT-PCR. Scale bar: 100 μm. Results represent mean ± SEM. * *p* < 0.05, Normal compared with DSS; ^#^ *p* < 0.05, DSS compared with DSS + Corylin (L); ^¥^ *p* < 0.05, DSS compared with DSS + Corylin (H); ^§^ *p* < 0.05, DSS + Corylin (L) compared with DSS + Corylin (H). DSS, dextran sodium sulfate; LGR5, leucine-rich repeat-containing G-protein-coupled receptor 5; PCNA, proliferating cell nuclear antigen; BrdU, bromodeoxyuridine (5-bromo-2′-deoxyuridine; DAPI, 4′,6-diamidino-2-phenylindole.

LGR5 and CD44 are potential markers for isolating colon cancer stem cells (CSCs), which promote proliferation and tumor formation. To understand whether cancer stem cells and intestinal epithelial cells are involved in DSS-induced colitis, LGR5, CD44, Ki67, PCNA, BrdU, and Cyclin D1 were examined in the colon (Figure 4A). To further confirm the LGR5 expression results in colitis, *Lgr5*, *Olfm4*, and *Cyclin* D1 mRNA levels were assessed in the colon of DSS-induced colitis mice (Figure 4B). Corylin significantly decreased colon cancer stem cell and intestinal epithelial cell proliferation in DSS-induced colitis.

### 2.5. Corylin Attenuated AOM/DSS-Induced Colitis Signs and Intestinal Damage

**Figure 5:**
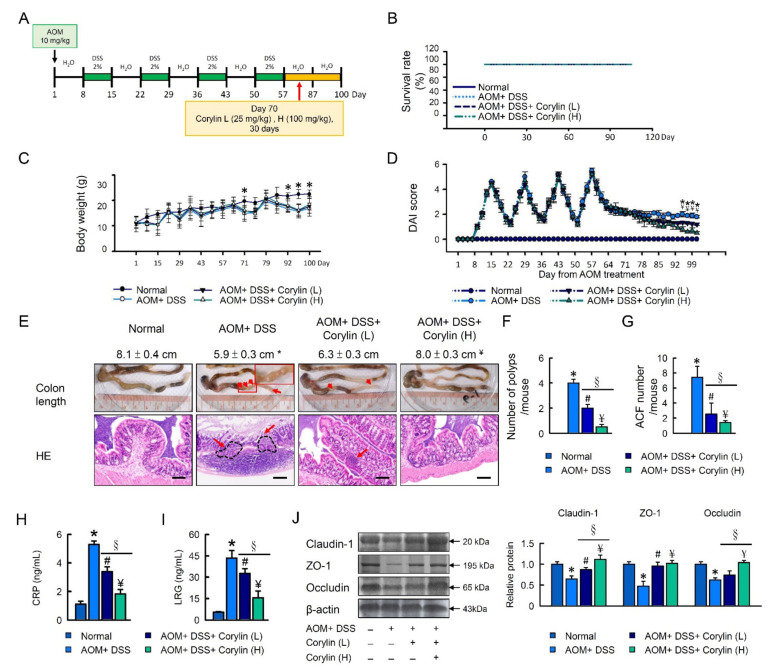

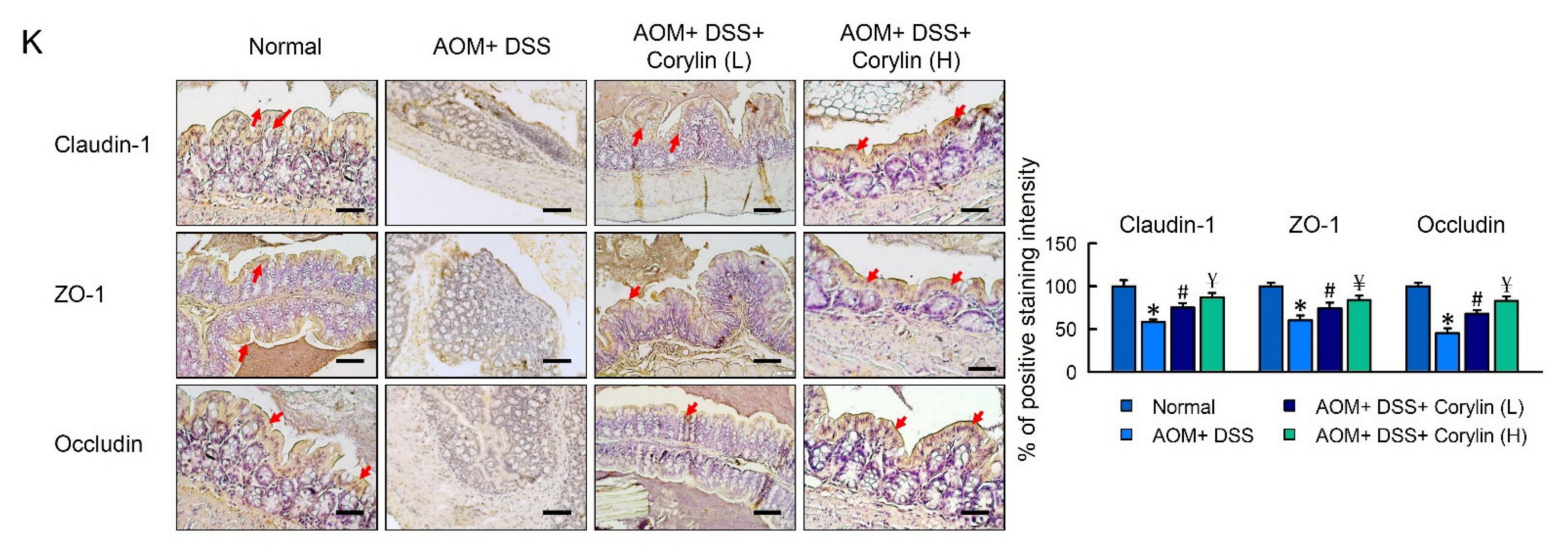
Corylin ameliorates AOM/DSS-induced-colitis-associated colorectal cancer in mice. (**A**) Schematic of the recurring model of AOM/DSS-induced-colitis-associated colorectal cancer. (**B**) Survival rate in experimental mice. (**C**) Body weight curves of WT mice treated with AOM/DSS and corylin. (**D**) Disease activity index (DAI) score in experimental mice. (**E**) Representative images of hematoxylin and eosin staining and of colon tissues of mice. Red arrows highlight the damage. Scale bar: 100 μm. Colon length from experimental mice. (**F**) Number of polyps, and (**G**) ACF in WT mice treated with AOM/DSS and corylin. (**H**) CRP and (**I**) LRG levels in experimental mice. (**J**) Western blot analysis of claudin-1, ZO-1, occludin, and β-actin (loading control) in colon homogenates. Right graph indicates quantification relative to β-actin. (**K**) Expression analysis by immunohistochemical staining of claudin-1, ZO-1, and occludin in mice after the indicated treatment. Tissue staining, as quantified by either stain intensity, is represented on the left-hand vertical axis in each graph. Results represent mean ± SEM. * *p* < 0.05, Normal compared with AOM/DSS; ^#^ *p* < 0.05, AOM/DSS compared with AOM/DSS + Corylin (L); ^¥^ *p* < 0.05, AOM/DSS compared with AOM/DSS + Corylin (H); ^§^ *p* < 0.05, DSS + Corylin (L) compared with DSS + Corylin (H). AOM, azoxymethane; DSS, dextran sodium sulfate; ACF, aberrant crypt foci; ZO-1, zonula occludens-1; CRP, C-reactive protein; LRG, leucine-rich alpha 2 glycoprotein.

In this study, we first investigated the effect of corylin on alleviating CAC. To evaluate whether corylin can mitigate or prevent CAC, we administered AOM/DSS to three groups of C57BL/6 mice and treated them with vehicle or corylin (25 or 100 mg/kg) every day for 30 days. The fourth group of mice was untreated (no AOM, DSS, or corylin) and served as the control group (*n* = 5) (Figure 5A). There were significant differences in the body weight, colon length, clinical signs, number of polyps, and ACF, but no differences in survival rate were observed following the administration of corylin to the preclinical cancer AOM/DSS mice (Figure 5B–F). Additional, serum CRP and LRG were significantly reduced in DSS-induced colitis mice treated with corylin (Figure 5H,I). The colons showed more severe inflammation with more cryptitis/crypt abscess formation, erosion, or ulceration in AOM/DSS-induced CAC mice compared with normal mice (Figure 5B–I). Meanwhile, histological examination exhibited increased number of polyps, ACF, neoplastic glands, colonic epithelial cell disorder, and inflammation as well as larger cell nuclei in the model group, whereas these effects were markedly ameliorated by corylin treatment (Figure 5E–I).

The expression levels of claudin-1, ZO-1, and occludin were significantly decreased in the AOM/DSS treatment group. Compared with the AOM/DSS treatment group, the levels of claudin-1, occludin, and ZO-1 were significantly increased in the corylin treatment groups. Furthermore, as shown in Figure 5J,K, corylin at a dose of 100 mg/kg (H) significantly increased the expression levels of claudin-1 and occludin, which were even higher than the effects produced by corylin at a dose of 25 mg/kg (L). Therefore, we propose that the effects of corylin on AOM/DSS-induced colitis mice are related to the regulation of the colonic mucosal barrier function, where the expression levels of claudin-1, ZO-1, and occludin play important roles in maintaining the intestinal mucosal barrier function (Figure 5J,K). However, corylin administration could dose-dependently lower colon damage in AOM/DSS-induced CAC mice (Figure 5).

### 2.6. Corylin Attenuated the TLR4/p38/AP-1 Signal Pathway and Inflammation in AOM/DSS-Induced-Colitis-Associated Colorectal Cancer (CAC) Mice

**Figure 6:**
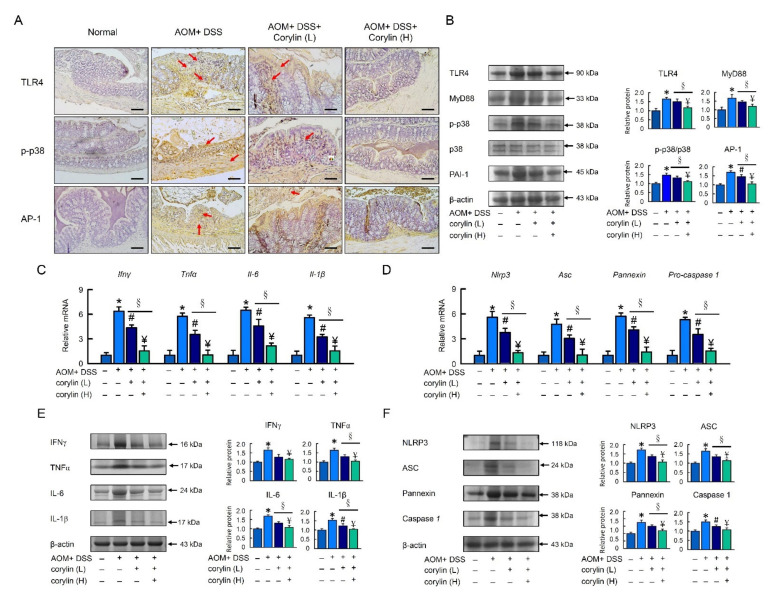
Corylin improves the TLR4 signaling pathway in AOM/DSS-induced-colitis-associated colorectal cancer mice. (**A**) Expression analysis by immunohistochemical staining of TLR4, p-p38, and AP-1 in mice after the indicated treatment. Scale bar: 100 μm. (**B**) Western blot analysis of TLR4, MyD88, p-p38, AP-1, and β-actin (loading control) in colon. (**C**,**D**) mRNA expression of *Ifnγ*, *Tnf-α*, *Il-6*, *Il-1β*, *Nlrp3*, *Asc*, *Pannexin*, and *Pro-caspase 1* as determined by qRT-PCR. (**E**,**F**) Western blot analysis of IFNγ, TNF-α, IL-6, IL-1β, NLRP3, ASC, Pannexin, and Caspase 1, and β-actin (loading control) in colon homogenates. Results represent mean ± SEM. * *p* < 0.05, Normal compared with AOM/DSS; ^#^ *p* < 0.05, AOM/DSS compared with AOM/DSS + Corylin (L); ^¥^ *p* < 0.05, AOM/DSS compared with AOM/DSS + Corylin (H); ^§^ *p* < 0.05, DSS + Corylin (L) compared with DSS + Corylin (H). AOM, azoxymethane; DSS, dextran sodium sulfate; TLR4, Toll-like receptor 4; MyD88, myeloid differentiation primary response 88; AP-1, activator protein 1; *Ifnγ*, interferon gamma; *Tnf-α*, tumor necrosis factor-α; *Il-6*, interleukin-6; *Nlrp3*, NACHT, LRR, and PYD domains-containing protein 3.

Progressively activated TLR4 encourages the proliferation of colon cancer cells and extricates cancer cells from death. As shown in Figure 6A, AOM/DSS immunostaining showed the highest level of TLR4, p-p38, and AP-1 in colon tissue. However, we found that the TLR4, p-p38, and AP-1 levels following corylin treatments was significantly lower than that following AOM/DSS treatment. We also demonstrated that AOM/DSS increased the protein expression of TLR4, p-p38, and AP-1, whereas corylin treatments inhibited the overexpression of TLR4, p-p38, and AP-1 (Figure 6B).

To investigate the involvement of chronic inflammation in AOM/DSS-treated mice and elucidate the molecular mechanism of corylin, we evaluated the effects of corylin on inflammatory cytokines and NLRP3 inflammasome expression in the AOM/DSS-induced CAC mouse model. As shown in Figure 6C–F, the AOM/DSS group demonstrated a marked difference compared with the control group. However, corylin inhibited the mRNA expression and protein levels of *Ifnγ*, *Tnf-α*, *Il-6*, *Il-1β*, *Nlrp3*, *Asc*, *Pannexin*, and *Pro-caspase 1* (Figure 6C–F) in the tumor tissues of AOM/DSS-induced CAC mice. Administration of 100 mg/kg (high dose) corylin significantly reduced the inflammatory response, showing a more beneficial effect than 25 mg/kg corylin in AOM/DSS-induced CAC mice.

### 2.7. Effects of Corylin on Macrophage Polarization in AOM/DSS-Induced CAC Mice

**Figure 7:**
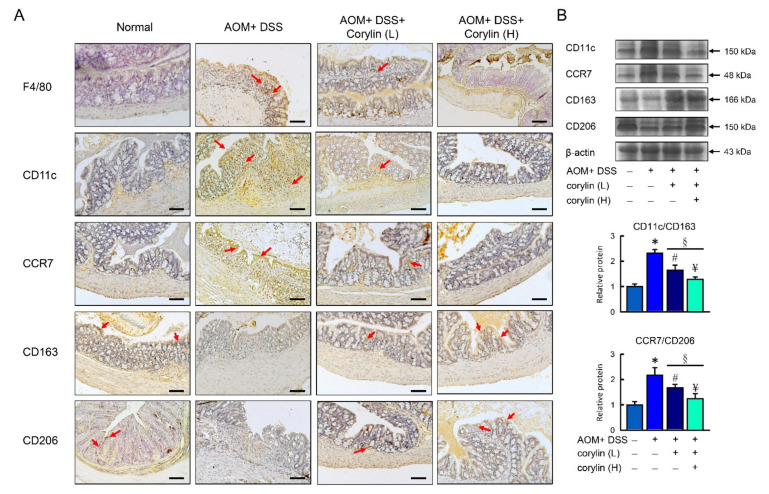
Corylin alters macrophage polarization in AOM/DSS-induced colitis mice. (**A**) Representative F4/80, CD11c, CCR7, CD163, and CD206 staining of colon. Red arrows highlight the positive staining. Scale bar: 100 μm. (**B**) Above: Western blot analysis of CD11c, CCR7, CD163, CD206, and β-actin (loading control) in colon. Below: Quantification of CD11c, CCR7, CD163, and CD206 protein levels in graphs indicating quantification ratio of CD11c and CD163 as well as ratio of CCR7 and CD206 levels. Results represent mean ± SEM. * *p* < 0.05, Normal compared with DSS; ^#^ *p* < 0.05, AOM/DSS compared with AOM/DSS + Corylin (L); ^¥^ *p* < 0.05, DSS compared with AOM/DSS + Corylin (H); ^§^ *p* < 0.05, DSS + Corylin (L) compared with DSS + Corylin (H). AOM, azoxymethane; DSS, dextran sodium sulfate.

We further analyzed the functional role of corylin in AOM/DSS-induced CAC mice. M1 macrophages can contribute to the pathogenesis of colitis, and M2-like macrophages are known to promote tissue repair, remodel immune regulation, and promote tumors. We analyzed the F4/80 (macrophage), CD11b, CCR7 (M1 markers), CD163, and CD206 (M2 markers) macrophage markers in colon tissue. According to Figure 7A,B, AOM/DSS-treated mice exhibited an increase in the M1-like phenotype and a reduction in the M2-like phenotype compared with the control mice with colon cancer. In addition, corylin significantly increased the expression of CD163 and CD206 (M2 markers) and reduced the expression of CD11b and CCR7 (M1 markers) as well as reversing the ratio of M1/M2 macrophages in AOM/DSS-treated mice (Figure 7A,B).

### 2.8. Effect of Corylin on Stem-like Cell and Intestinal Epithelial Cell Proliferation in AOM/DSS-Induced Colitis Mice

**Figure 8:**
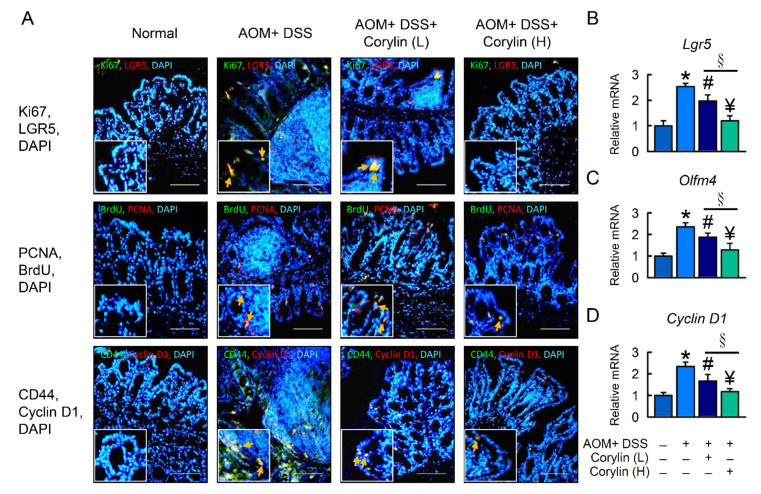
Corylin affects LGR5 and proliferation in AOM/DSS-induced-colitis-associated colorectal cancer mice. (**A**) Top line: Double immunofluorescence staining of Ki67 (green), LGR5 (red), and DAPI (blue) in colon. The staining for Ki67/LGR5 from colon expressing both markers is identified. Middle line: Double immunofluorescence staining of BrdU (green), PCNA (red), and DAPI (blue) in colon. The staining for BrdU/PCNA from colon expressing both markers is identified. Bottom line: Double immunofluorescence staining of endogenous CD44 (green), cyclin D1 (red), and DAPI (blue) in colon. The staining for CD44/cyclin D1 from colon expressing both markers is identified. DAPI (blue). Yellow arrows highlight positive staining. Scale bar: 100 μm. (**B**–**D**) mRNA expression of *Lgr5*, *Olfm4*, and *Cyclin D1* as determined by qRT-PCR. Scale bar: 100 μm. Results represent mean ± SEM. * *p* < 0.05, Normal compared with AOM/DSS; ^#^ *p* < 0.05, AOM/DSS compared with AOM/DSS + Corylin (L); ^¥^ *p* < 0.05, AOM/DSS compared with AOM/DSS + Corylin (H); ^§^ *p* < 0.05, DSS + Corylin (L) compared with DSS + Corylin (H). AOM, azoxymethane; DSS, dextran sodium sulfate; LGR5, leucine-rich repeat-containing G-protein-coupled receptor 5; PCNA, proliferating cell nuclear antigen; BrdU, bromodeoxyuridine (5-bromo-2′-deoxyuridine; DAPI, 4′,6-diamidino-2-phenylindole.

To investigate the possibility that corylin affects CAC, we analyzed cancer stem cell and intestinal epithelial cell proliferation in the AOM/DSS mice. LGR5 and CD44 staining in colonic crypt cells and tumor epithelia showed that AOM/DSS induced tumor formation. In assessing the effect of AOM/DSS on cell proliferation, Ki67, PCNA, BrdU, and Cyclin D1 expression was identified in the colonic crypt cells and tumor epithelia by immunofluorescence staining. Treatment with 100 mg/kg corylin in the AOM/DSS mice led to significantly decreased cancer stem cell and intestinal epithelial cell proliferation, including LGR5, CD44, Ki67, PCNA, BrdU, and Cyclin D1 levels, compared with the AOM/DSS group (Figure 8A). Indeed, mRNA expression of *Lgr5*, *Olfm4*, and *Cyclin D1* was increased in the AOM/DSS group, and corylin reversed that effect in the colon of mice (Figure 8B–D). In summary, the data indicate that corylin inhibited AOM/DSS-induced development of CAC.

### 2.9. Corylin Improved Gut Microbiota Diversity in AOM/DSS-Induced-Colitis-Associated Colorectal Cancer Mice

**Figure 9:**
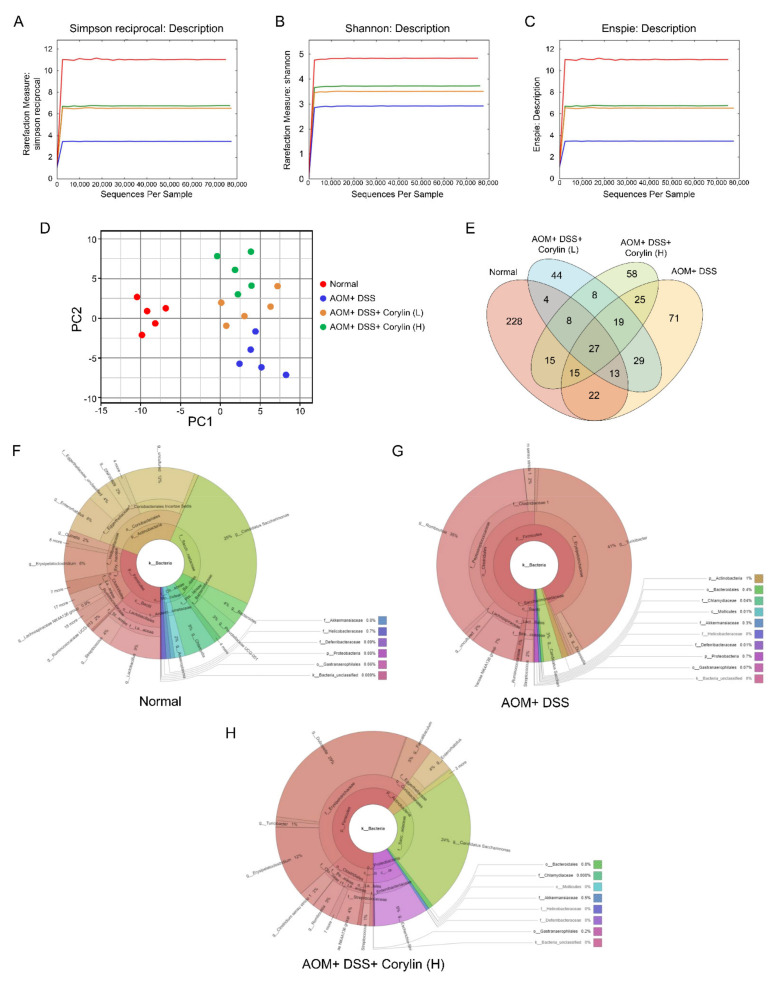
Corylin improved gut microbiota diversity in AOM/DSS-induced-colitis-associated colorectal cancer mice. (**A**) Simpson reciprocal, (**B**) Shannon index, and (**C**) ENSPIE represent the alpha diversity indices. Simpson reciprocal, Shannon index, and ENSPIE represent species diversity, abundance, and evenness, respectively. (**D**) PCA plot based on bacterial sequence abundance in fecal content. Axes correspond to principal components 1 (x-axis) and 2 (y-axis). (**E**) The distribution of operational taxonomic units (OTUs) among four different treatment groups is represented via the Venn diagram. (**F**–**H**) In the interactive html result, circles show different classification levels from the phylum to the genus (inside to outside) in the community. AOM, azoxymethane; DSS, dextran sodium sulfate.

In our study, Illumina MiSeq (16S rRNA gene) was employed to characterize the overall pattern of the gut microbiota community in AOM/DSS- and corylin-treated mice. In order to demonstrate the bacterial diversity, richness, abundance, and structural differences in each group, alpha diversity indices were evaluated. The alpha diversity parameters were determined using the Simpson reciprocal, the Shannon index, and ENSPIE (Figure 9A–C). In the AOM/DSS group, the relative scores of alpha diversity indices were reduced compared to the normal group. However, corylin treatment evidently enhanced the alpha diversity indices in comparison with the AOM/DSS group (Figure 9A–C). Altogether, our findings revealed that the alpha diversity indices improved after corylin treatment, especially at the high dose. To elucidate the structural variability among the different treatment groups, we conducted a principal component analysis (PCA) (Figure 9D). Our findings showed that AOM/DSS subjects were clustered separately from the control group. However, the corylin treatment group, particularly at a higher dose, and the control group clustered more closely relative to the AOM/DSS group. These data suggest that the normal and corylin-treated groups were more similar compared to the AOM/DSS group. We also assessed the shared and group-specific OTUs across each group (Figure 9E) and found 27 OTUs were shared by all the treatment groups. Each leaflet depicts the number of OTUs in the respective group (Figure 9E).

Next, we investigated the hierarchical relationship of all the gut microbiota. In the interactive html result, the circles show the different classification levels from the phylum to the genus (inside to outside) in the community, and the area of the sector shows the respective proportion of different OTU annotation results (Figure 9F,G). Corylin treatment reversed the dysbiosis pattern effectively at the high dose in the AOM/DSS + corylin (H) group compared to the AOM/DSS group (Figure 9F–H). It also greatly improved gut microbiota composition, and flora mostly returned to the normal state at a high dose of corylin.

### 2.10. Corylin Altered Intestinal Microbial Composition in AOM/DSS-Induced-Colitis-Associated Colorectal Cancer Mice

**Figure 10:**
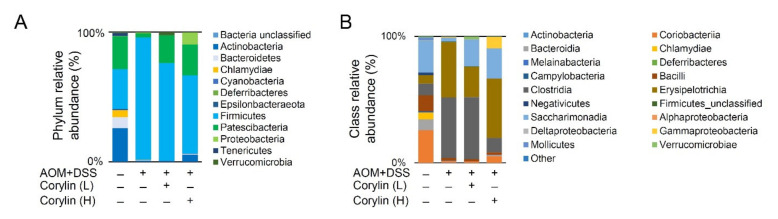

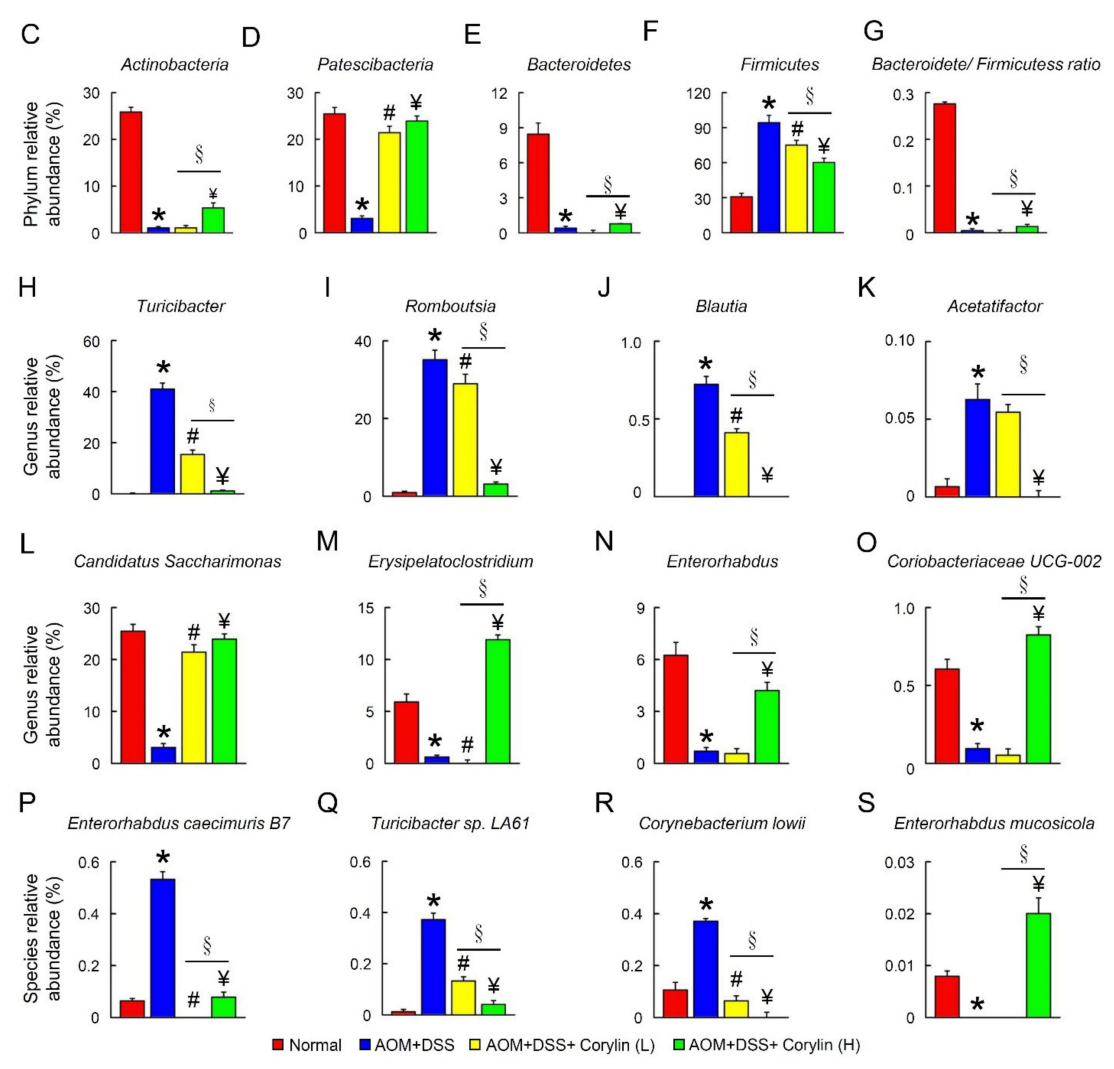
Corylin alters the intestinal microbial composition in AOM/DSS-induced-colitis-associated cancer mice. Microbial community bar plot by (**A**) phylum and (**B**) class relative abundance (%). Phylum levels of (**C**) *Actinobacteria,* (**D**) *Patescibacteria*, (**E**) *Bacteroidetes*, (**F**) *Firmicutes*, and (**G**) *Bacteroidetes/Firmicutes* ratio. Genus levels of (**H**) *Turicibacter*, (**I**) *Romboutsia*, (**J**) *Blautia,* (**K**) *Acetatifactor,* (**L**) *Candidatus Saccharimonas*, (**M**) *Erysipelatoclostridium*, (**N**) *Enterorhabdus*, and (**O**) *Coriobacteriaceae UCG-002*. Species abundance of (**P**) *Enterorhabdus caecimuris B7*, (**Q**) *Turicibacter* sp. *LA61*, (**R**) *Corynebacterium lowii*, and (**S**) *Enterorhabdus mucosicola*. Values express the mean ± standard error. The number of mice per experiment is *n* = 5. * *p* < 0.05, Normal compared with AOM/DSS; ^#^ *p* < 0.05, AOM/DSS compared with AOM/DSS + Corylin (L); ^¥^ *p* < 0.05, AOM/DSS compared with AOM/DSS + Corylin (H); ^§^ *p* < 0.05, DSS + Corylin (L) compared with DSS + Corylin (H). AOM, azoxymethane; DSS, dextran sodium sulfate.

According to the species annotation, the statistical number of sequences of every sample at each classification level (phylum, class, genus, and species) was calculated. We created a bar plot to present the results. All sequences were classified and identified from phylum to species level (Figure 10). The 16S rRNA profiles of each experimental group in the colon were very dissimilar, even in phylum- and class-level distributions (Figure 10A,B). AOM/DSS reduced *Actinobacteria, Patescibacteria*, *Bacteroidetes*, *Bacteroidetes/Firmicutes* ratio, *Candidatus Saccharimonas*, *Erysipelatoclostridium*, *Enterorhabdus*, *Coriobacteriaceae UCG-002*, and *Enterorhabdus mucosicola* (Figure 10C–E,G,L–O,S) and increased the relative abundance of *Firmicutes*, *Turicibacter*, *Romboutsia*, *Blautia, Acetatifactor, Enterorhabdus caecimuris B7*, *Turicibacter* sp. *LA61*, and *Corynebacterium lowii* (Figure 10H–K,P–R). At the lower dose in AOM/DSS + corylin (L), variation persisted as compared to the AOM/DSS + corylin (H) and normal groups. Furthermore, at the phylum, class, genus, and species levels, the bacterial composition varied in the AOM/DSS treatment group compared to the normal group. The effects were improved following treatment with the high corylin dose.

## 3. Discussion

Corylin is a natural flavonoid compound that is extracted and purified from *Psoralea corylifolia* L. (Fabaceae) [23]. The herb is commonly used in traditional Chinese medicine for the treatment of different diseases, such as cardiovascular diseases [24], and as an osteoclast inhibitor against osteoporosis [25,26]. Among the natural products from *P*. *corylifolia* L., corylin is the main flavonoid and shows several therapeutic effects, such as anti-inflammatory, anti-oxidation, and anti-cancer effects for hepatocellular carcinoma [23], as well as playing a role in the treatment of colorectal cancer (CRC) through inhibiting the STAT3 pathway [27]. Furthermore, corylin reportedly exerts anti-obesity effects through the browning of white adipocytes, activation of browning adipose tissue, and promotion of lipid metabolism [20]. This provoked our curiosity to explore the protective link between corylin and the gut flora as well as the molecular mechanisms of corylin that link the gut microbiota with the colonic macrophage polarization in a colitis-associated cancer (CAC) model.

The pathogenesis of inflammatory bowel disease (IBD) may be characterized by a loss of intestinal epithelial integrity and the dysregulation of several proteins, including transmembrane proteins, such as zona occludens (ZO)-1, occludin, claudins, and tight junctional molecules [28,29,30], in DSS-induced acute colitis mice [31]. Our results show that corylin significantly alleviated weight loss and the survival rate scores in DSS-induced colitis mice and reduced the infiltration of macrophages. In addition, corylin can inhibit macrophage M1 polarization and reduce the expression of the pro-inflammatory cytokines TNF-α, IL-1β, and IL-6. In this study, DSS could activate TLR4-mediated inflammatory pathways, such as the MyD88 and p38 phosphorylation cascade, to induce the production of inflammatory cytokines (IL-1β, IL-6, and TNF-α), which have a potential correlation with the expression of tight junction proteins [32]. This may partially explain the enhanced expression of ZO-1 and claudin-1, since the overproduction of IL-6 and TNF-α was considered to participate in enhancing intestinal epithelial permeability [33]. It has been demonstrated that increased levels of pro-inflammatory mediators, including IL-1β, IL-6, and TNF-α, in colon tissue were also highly elevated in response to invading bacteria and their products in IBD patients [34]. Furthermore, our results demonstrated that corylin treatment significantly inhibited LGR5/Olfm4 expression in the colon tissue. We found that corylin administration markedly reversed the increased macrophage infiltration and polarization in colon tissues in DSS-induced colitis mice. These anti-inflammatory properties led us to propose the potential role of corylin in reducing colon cancer stem cell proliferation and tumor formation. The colon remains the primary site of neoplasms in inflammatory bowel disease (IBD) patients today, and colorectal cancer accounts for approximately 10-15% of all deaths in IBD patients [35]. Patients with IBD colitis are six times more likely to develop colorectal cancer than the general population and have a higher frequency of multiple synchronous colorectal cancers [35]. Among these risk factors, chronic inflammation in IBD-affected individuals contributes to the initiation and progression of colorectal cancer (CRC), and anti-inflammatory interventions can help to prevent the development of colitis-associated colorectal cancer (CAC) [36]. AOM and DSS are genotoxic and non-genotoxic colonic carcinogens, respectively. Their combination leads to colitis-related carcinogenesis development through inflammation of colon cells [37]. The importance of aberrant crypt foci (ACF) is known as a good predictor of synchronous and metachronic adenoma or other polyps whose removal reduces the risk of CRC [38]. ACF as the earliest identifiable biomarker of colorectal neoplasia and response to therapy. In the present study, we investigated the anti-CAC potential of corylin and the underlying mechanism. In contrast to hyperplastic ACF and proliferation (Ki67 and PCNA positivity), in dysplastic ACF foci extend to the epithelial surface. In contrast, dietary supplementation of high dose corylin for 30 days caused a dose-dependent reduction in the number of histologically confirmed dysplastic ACF within the colon of AOM/DSS-treated mice.

These results showed that orally administered corylin markedly inhibited AOM/DSS-induced CAC in mice, as evidenced by the tumor number, size, ACF, colon polyps and histopathological examination. In this study, AOM/DSS treatment of normal mice increased pro-inflammatory cytokines and inflammasome gene expression, with the observation of shorter colon lengths, and epithelial cell proliferation, potentially associated with this. However, administration of corylin to AOM/DSS-induced CAC mice showed the opposite pattern regarding the production of those cytokines, which was probably associated with the recovered colon length and reduced number of stem-like cells and epithelial cell proliferation, suggesting the potential anti-inflammatory properties of corylin in the AOM/DSS model. Furthermore, corylin ameliorates AOM/DSS-induced colonic carcinogenesis in mice, probably due to the breakdown of the imbalanced M1/M2 macrophage ratio; administration of corylin ameliorates this symptom by restoring the homeostasis of the macrophage polarization.

In this study, it appears that corylin’s anti-carcinogenic properties are most likely due to its effects on multiple molecular targets, such as macrophage polarization and epithelial cell proliferation. Macrophages are the most common non-neoplastic cells in human tumors, including colorectal cancer [38,39]. Traditionally, macrophages have been categorized as either pro-inflammatory M1-like macrophages or anti-inflammatory M2-like macrophages. In particular, tumor-associated macrophages, exposed to multiple polarization stimuli in the tumor microenvironment, exist between these two extremes of macrophage polarization [40,41]. The results of this study show that the administration of corylin significantly attenuated tumorigenesis. After administration of corylin, the relative abundance of M1 macrophages and intestinal epithelial cell proliferation were decreased, which was associated with the lower tumor burden. Corylin increased the levels of the colon tight junction markers ZO-1, claudin, and occludin in CAC mice by repairing TLR4 signaling and decreasing the phosphorylation of p38 and downregulation of inflammasome genes, thus affecting tumor progression. Our results indicate that corylin improves gut barrier integrity, reduces inflammatory response, decreases TLR4 signaling, and decreases inflammation in AOM/DSS-affected mice. The beneficial effects induced by corylin treatment may therefore be attributed to alterations in the gut microbiota. The anti-tumor effect of corylin was also evidenced by its ability to improve the intestinal microbiota diversity and composition. Microbiota were integrated in the framework of CAC, and it seems that dysbiosis and subsequent immunological responses facilitate CAC carcinogenesis [42,43,44]. The potential bacterial and carcinogenic metabolites, intraluminal events, and inflammatory pathways are being subjected to research, as manipulation of the microbiota may change the course of tumorigenesis [44].

In our study, we have characterized how tumorigenesis was suppressed in the corylin treatment groups, where the relative signaling of the TLR4 cascade and gut barrier permeability were also repaired and the abundance of the phylum *Bacteroidetes* and *Firmicutes* ratio was relatively somewhat maintained in AOM/DSS mice. In an American patient cohort, an increased abundance of *Firmicutes* was observed compared with non-adenoma subjects in adenoma biopsies [45]. The *Firmicutes*/*Bacteroidetes* (F/B) ratio is widely accepted as having an important influence in maintaining normal intestinal homeostasis. An increased or decreased F/B ratio is regarded as dysbiosis, whereby the former is usually observed with obesity and has been associated with inflammatory bowel disease (IBD) [46]. Our results support previous results showing that the overall gut microbial community plays an important role in colon tumorigenesis. However, these findings should not be interpreted as assessing whether the change in *Firmicutes* is a direct result of AOM/DSS stimuli or an indirect effect of the decrease in tumorigenesis from corylin’s contribution. *Firmicutes*, which, as part of the gut microbiome, has been shown to be involved in energy resorption [47], has highly diverse phenotypic characteristics. Members of the phylum display a disparate distribution, in which some species are enriched in the tumor tissue whereas others inhabit a healthy gut [48]. Microbial alpha diversity is a measure quantifying the overall community complexity and is known to be affected by gut physiology [49]. We observed increasing microbial diversity, measured by Shannon’s diversity index, following corylin treatment of the colon in mucosal samples from AOM/DSS mice. In addition, our findings also demonstrated that bacteria belonging to the same taxonomic clade can display distinct functional roles in the gut microenvironment depending on their functional repertoire, including toxins, virulence factors, and metabolite factors that promote interactions between the bacteria and their microenvironment.

In a previous study, using a magnetically controlled sampling capsule endoscope (MSCE), a dramatic decrease in the abundance of *Bacteroidetes*, *Patescibacteria*, and *Actinobacteria* was detected in the antibiotic-induced diarrhea model [50]. In contrast, the microbiota composition in signet-ring cell carcinoma (SRCC) tumor samples was significantly enriched in the phyla *Fusobacteria, Bacteroidetes*, and *Patescibacteria* [51]. The phylum *Patescibacteria* was enriched throughout the luminal content in the small intestine when compared with that of the colon of preterm lambs [52]. These results suggest that the abundance of bacteria, which depends on disease procession, is not typically associated with a healthy gut microbiome, but does commonly co-exist with commensals observed in different disease states. Interestingly, we observed that the phylum *Patescibacteria* was most abundantly involved in corylin treatment in AOM/DSS mice. *Patescibacteria* lack a tricarboxylic acid (TCA) cycle and most elements of the electron transfer chain, suggesting that they are strict anaerobic fermenters [53]. Based on their limited biosynthetic capabilities and reduced repertoire of DNA repair genes, it has been postulated that members of *Patescibacteria* may illustrate a genomic reduction process towards an ectosymbiotic lifestyle [54]. The tricarboxylic acid (TCA) cycle and carbon metabolism play an important role in tumors. Our study revealed a possible connection between the TCA cycle and carbon metabolism and corylin, showing a TCA-cycle- and carbon-metabolism-related *Patescibacteria* signature, which could show a negative trend in AOM/DSS mice.

Changes in the microbiota have been observed in previous studies; decreased bacterial diversity and increased bacterial instability were verified in patients with IBD compared with healthy individuals [55,56]. In our results, at the genus level, AOM/DSS-treated mice had a higher relative abundance of *Turicibacter*, *Romboutsia*, *Blautia*, and *Acetatifactor* than the normal controls. Moreover, a decrease in the relative abundance of *Candidatus saccharimonas*, *Erysipelatoclostridium*, *Enterorhabdus*, and *Coriobacteriaceae* UCG-002 was observed. *Blautia* are anaerobic bacteria with probiotic characteristics that occur widely in the feces and intestines of mammals. Evidence indicates the importance of the survival and evolution of *Blautia* in the gut and other microenvironments [57]. *Blautia* was the only gut microbe significantly and inversely associated with visceral fat area (VFA) regardless of sex [58]. The abundances of *Blautia* and *Ruminococcus* in colorectal neoplasm patients were higher than in healthy controls [59]. *Blautia* were significantly increased in a colorectal cancer group [60]. On the other hand, the microbial *Erysipelatoclostridium* was identified in the early detection of colorectal cancer across populations [61]. Vitamin B2 can decrease the abundance of *Erysipelatoclostridium* [62]. As shown in our results, at the genus level, the relative abundance of *Erysipelatoclostridium* was enriched in AOM/DSS mice treated with corylin. Therefore, we suspect that *Erysipelatoclostridium* is a beneficial bacterium in the gut. However, the specific role of these gut microbiota needs to be further investigated. Taken together, the present study suggests that corylin may protect the intestinal barrier from AOM/DSS damage by altering the *Blautia*/*Erysipelatoclostridium* ratio, as well as modifying the levels of other specific bacterial species.

In summary, we found that corylin administration during the late stage is effective at reducing tumorigenesis signaling. This was associated with a decrease in select M1/M2 macrophage polarization in the colon tissue linked to CAC. Overall, these data support the development of therapeutic strategies to target macrophages in CAC, and the results provide a theoretical basis for understanding the relationship between the intestinal barrier function and gut microbiota modulated by corylin, although further investigations, based on metabolomics, should be conducted in future studies.

## 4. Materials and Methods

### 4.1. Animals

Eight-week-old male wild-type C57BL/6J mice were purchased from the National Laboratory Animal Center (Taiwan) and housed with free access to sterile chow diets and water under a standard temperature (23~25 °C) and humidity-controlled (40–60%) environment. These studies were approved by the Animal Care and Use Committee at the Chang Gung University (CGU106-189).

### 4.2. DSS-Induced Colitis Model

Four groups (*n* = 5) of eight-week-old male wild-type C57BL/6J mice were fed dextran sulfate sodium (DSS, 42867, Merck, USA) dissolved in sterile distilled water (4%) for up to 12 days and fed corylin (PHL83287, Merck, USA) at 25 mg/kg/day (L) and 100 mg/kg/day (H) orally from day 3 for 10 days [20]. The DSS model was created using 4% DSS treatment, as described previously [63].

### 4.3. AOM/DSS-Induced Colon Cancer Model

The AOM/DSS model was created by injecting 10 mg/kg body weight of AOM once, with four further cycles of 2% DSS treatment, as described previously [62]. Four groups (*n* = 5) of eight-week-old male wild-type C57BL/6J mice were first induced to form AOM/DSS models and then fed 25 mg/kg/day (L) and 100 mg/kg/day (H) corylin from day 70 to day 100 (30 days). Body weight and survival were recorded daily during experiments. Animals were euthanized with CO_2_ inhalation followed by cervical dislocation at the end of the experiments. Colon length was measured, and colon tissues were fixed in PBS-buffered 10% formalin for histopathology or processed for molecular biology studies.

### 4.4. HE, Immunohistochemical, and Immunofluorescence Staining

Colonic tissues were fixed in 10% formalin and then embedded in paraffin wax after dehydration with xylene and alcohol. The tissues were used to cut 5-micrometer sections, which were then stained with hematoxylin and eosin according to the standard protocols. The slides were then incubated with ZO-1 (ab216880, Abcam, Trumpington, Cambridge, UK), claudin-1 (ab15098, Abcam, Trumpington, Cambridge, UK), occludin (ab216327, Abcam, Trumpington, Cambridge, UK), TLR4 (ab13867, Abcam, Trumpington, Cambridge, UK), MyD88 (ab28763, Abcam, Trumpington, Cambridge, UK), p-p38 (ab178867, Abcam, Trumpington, Cambridge, UK), F4/80 (Ab6640, Abcam, Trumpington, Cambridge, UK), CD11c (ab52632, ab11029, Abcam, Trumpington, Cambridge, UK), CCR7 (ab32527, Abcam, Trumpington, Cambridge, UK), CD163 (ab182422, Abcam, Trumpington, Cambridge, UK), CD206 (ab64693, Abcam, Trumpington, Cambridge, UK), Ki67 (ab15580, Abcam, Trumpington, UK), LGR5 (ab219107, ab75850, Abcam Trumpington, Cambridge, UK), BrdU (ab6326, Abcam, Trumpington, Cambridge, UK), PCNA (ab29, Abcam, Trumpington, Cambridge, UK), CD44 (ab189524, Abcam, Trumpington, Cambridge, UK), cyclin D1 (ab16663, Abcam, Trumpington, Cambridge, UK), and AP-1 (MFCD02095845, Merck, Kenilworth, NJ, USA) primary antibodies overnight night at 4 °C. Secondary antibody was conjugated with HRP-conjugated anti-rabbit (Millipore, Burlington, MA, USA), anti-mouse (Millipore, Burlington, MA, USA), and anti-rat (Genetex, Alton Pkwy Irvine, CA, USA) secondary antibodies, followed by incubation in DAB peroxidase solution (Millipore, Burlington, MA, USA) and subsequent counterstaining with hematoxylin (Millipore, Burlington, MA, USA). Sections were incubated with the secondary antibody (Alexa Fluor 488, Alexa Fluor 633, anti-mouse, or anti-rabbit) (Thermo Fisher Scientific, Waltham, MA, USA) for immunofluorescence and DAPI (Thermo Fisher Scientific, Waltham, MA, USA) for nuclear staining. Images were obtained using Olympus cellSens (Olympus, Tokyo, Japan).

### 4.5. Western Blot

Colon tissues that had been stored at −80 °C were lysed in Thermo-Fisher Tissue Protein Extraction Reagent (Thermo Fisher Scientific, Rockford, IL, USA) containing a protease inhibitor cocktail (Roche Diagnostics, Basal, Switzerland). Protein concentrations were determined using the Bio-Rad Protein Assay (Bio-Rad, London, UK). Total extracted proteins were then incubated with tris-buffered saline containing 0.1% Tween-20 with 5% non-fat dried milk for 1 h, the blots were incubated with ZO-1 (ab216880, Abcam, Trumpington, Cambridge, UK), claudin-1 (ab15098, Abcam, Trumpington, Cambridge, UK), occludin (ab216327, Abcam, Trumpington, Cambridge, UK), TLR4 (ab13867, Abcam, Trumpington, Cambridge, UK), MyD88 (ab28763, Abcam, Trumpington, Cambridge, UK), p-p38 (ab178867, Abcam, Trumpington, Cambridge, UK), p38 (ab170099, Abcam, Trumpington, Cambridge, UK), CD11c (ab52632, ab11029, Abcam, Trumpington, Cambridge, UK), CCR7 (ab32527, Abcam, Trumpington, Cambridge, UK), CD163 (ab182422, Abcam, Trumpington, Cambridge, UK), CD206 (ab64693, Abcam, Trumpington, Cambridge, UK), β-actin (MAB1501, Millipore, Burlington, MA, USA), AP-1 (MFCD02095845, Merck, Kenilworth, NJ, USA), IFNγ (MM700B, Thermo Fisher Scientific, Rockford, IL, USA), TNF-α (#3707, Cell Signaling Technology, Beverly, MA, USA), IL-6 (ab259341, Abcam, Trumpington, UK), IL-1β (ab234437, Abcam, Trumpington, Cambridge, UK), NLRP3 (ab274490, Abcam, UK), ASC (sc-514414, Santa Cruz Biotechnology, Santa Cruz, California, USA), Pannexin (ab248078, Abcam, Trumpington, Cambridge, UK), and Caspase 1 (PA5-87536, Thermo Fisher Scientific, Rockford, IL, USA) antibodies overnight at 4^o^ C. Secondary antibody was conjugated with HRP-conjugated anti-rabbit (Millipore, Burlington, MA, USA), anti-mouse (Millipore, Burlington, MA, USA), and anti-rat (Genetex, Alton Pkwy Irvine, CA, USA) secondary antibody. Quantifications were performed using Image J.

### 4.6. Quantitative Real-Time Reverse Transcription Polymerase Chain Reaction (qRT-PCR) Assay

Total RNA was extracted from colon tissue using Trizol reagent (Invitrogen, Carlsbad, CA, USA). Complementary DNA (cDNA) was synthesized using the High-Capacity cDNA Reverse Transcription Kit (Thermo Fisher Scientific, Rockford, IL, USA). The cDNA was analyzed by quantitative real-time (qRT) PCR using specific primers (listed in Table 1) and the Power SYBR Green PCR Master Mix (Roche, Basel, Switzerland) in a LightCycler 1.5 (Roche, Basel, Switzerland). As a housekeeping gene, GAPDH was tested to verify the stability in this experimental model. Therefore, in this study, gene expression levels were normalized to those of GAPDH. The primer sequences used in this study are shown in Table 1.

### 4.7. Extraction of Genome DNA

Following euthanasia, feces from the colon were removed, snap-frozen, and stored at −80 °C until analysis. Total genome DNA from feces was extracted using the CTAB/SDS method [64]. DNA concentration and purity were monitored on 1% agarose gels. According to the concentration, DNA was diluted to 1 ng/uL using sterile water.

### 4.8. Amplicon Generation

Amplification of the 16S rRNA V3–V4 (341F-805R) hypervariable region was carried out using the 16S V3 (5′-CCTACGGGNGGCWGCAG-3′) and 16s_illumina_V4R (5′-GACTACHVGGGTATCTAATCC-3′) with added Illumina adapter overhang nucleotide sequences. The 16S/18S rRNA genes were amplified using specific primers with barcodes. All PCR reactions were carried out in 25-microliter reactions with 0.5 μL of KAPA^®^ High-Fidelity PCR Master Mix (KAPA BIOSYSTEMS), 0.5 μM of forward and reverse primers, and approximately 1 ng template DNA. The main tool of the workflow was QIIME, which contained multiple analysis process programs [65].

The 16S rRNA sequences were compared for similarity with the reference species of bacteria using the NCBI nucleotide database and the PhiX Control Library. To begin, sequences were clustered into operational taxonomic units (OTUs) using mothur v.1.39.5 with 97% identity. Chimera sequences were identified using UCHIME v4.2 (with reference data: GOLD database) and an OTU table was made [66]. We analyzed both the common and unique information for different samples (groups) and reported the results in a Venn diagram (VennDiagram v1.6.17). Microbial diversity can be evaluated within a community (alpha diversity) or between a collection of samples (beta diversity). Three different metrics were calculated to evaluate the alpha diversity: “Simpson”, “Shannon”, and “ENSPIE”. The level of alpha diversity in our analysis was calculated with QIIME. Then, we conducted a principal component analysis (PCA), which is a statistical procedure that uses an orthogonal transformation to convert a set of observations of possibly correlated variables into a set of values of linearly uncorrelated variables called principal components.

### 4.9. Measurement of Serum CRP Level

The serum level of C-reactive protein (CRP) was measured in mouse serum obtained at day 12 using a mouse CRP ELISA kit (R&D Systems, Minneapolis, MN, USA). ELISA assays were performed in accordance with the manufacturer’s instructions. The optical densities of the samples were determined using a microplate reader set at 450 nm.

### 4.10. Measurement of Serum LRG Level

The serum level of leucine-rich alpha2-glycoprotein (LRG) was measured in mouse serum obtained at day 12 using a mouse LRG Assay Kit (IBL, Fujioka, Japan). ELISA assays were performed in accordance with the manufacturer’s instructions. The optical densities of the samples were determined using a microplate reader set at 450 nm.

### 4.11. Measurement of DAI Score

To evaluate the severity of colitis, DAI scores were monitored daily. The DAI score was determined based on the methods of Friedman et al. [67] The DAI score was calculated as the sum of the weight loss score, the diarrheal score and the hematochezia score (Table 2).

### 4.12. Statistical Analysis

Data are expressed as the mean ± SEM. Unpaired, non-parametric Student’s *t*-tests with two-tailed distributions were performed to examine statistical differences between two experimental groups using Graph Pad Prism software 7.0 (Graph Pad Software, Inc., San Diego, CA, USA). *p*-values < 0.05 are regarded as statistically significant.

## Figures and Tables

**Table 1:** Oligonucleotide sequences for qRT-PCR.

Gene	Forward	Reverse
*IFNγ*	5′ cctcaaacttggcaatactc 3′	5′ agcaacaacataagcgtcat 3′
*TNFα*	5′ ttgacctcagcgctgagttg 3′	5′ cctgtagcccacgtcgtagc 3′
*IL-6*	5′ gtactccagaagaccagagg 3′	5′ tgctggtgacaaccacggcc 3′
*IL-1β*	5′ aacctgctggtgtgtgacgttc 3′	5′ cagcacgaggcttttttgttgt 3′
*Nlrp3*	5′ tgctcttcactgctatcaagccct 3′	5′ acaagcctttgctccagaccctat 3′
*Asc*	5′ gcaactgcgagaaggctatg 3′	5′ aagcatccagcactccgtc 3′
*Pannexin*	5′ tgaccacagacagcacttaag 3′	5′ cgtctgagagctccctggcg 3′
*Pro-caspase 1*	5′ cacagctctggagatggtga 3′	5′ ggtcccacatattccctcct3′
*Lgr5*	5′ cgttcgtaggcaacccttctctta 3′	5′ cgaggcaccattcaaagtcagtgt 3′
*Olfm4*	5′ ctgccagacaccacctttcc 3′	5′ ctcgaagtccagttcagtgtaag 3′
*Cyclin D1*	5′ gttcatttccaacccaccctc 3′	5′ agaaagtgcgttgtgcggtag 3′
*Gapdh*	5′ tcaccaccatggagaaggc 3′	5′ gctaagcagttggtggtgca 3′

*Il-1β*, interleukin-1β; *Tnfα*, tumor necrosis factor α; *Ifnγ*, interferon gamma; *Nlrp3*, NOD-, LRR-, and pyrin domain-containing protein 3; *Lgr5*, leucine-rich repeat-containing G-protein-coupled receptor 5; *Olfm4*, olfactomedin 4; *Gapdh*, glyceraldehyde-3-phosphate dehydrogenase.

**Table 2:** Scoring system of disease activity index ^a^.

Score	Weight Loss (%)	Stool Consistency	Hematochezia ^b^
0	None	Normal	Absence
1	0–10		
2	11–15	Loose stool ^c^	
3	16–20		
4	>20	Diarrhea	Presence

^a^ DAI = (score of weight loss) + (score of stool consistency) + (score of hematochezia). ^b^ The presence of gross blood in the stool or anus. ^c^ The formation of a stool that readily becomes paste on anus of mice.

## Data Availability

The data generated for this study are available from the corresponding author on reasonable request.
